# Supplementary material for: Retinal Vascular Occlusion after COVID-19 Vaccination: More Coincidence than Causal Relationship? Data from a Retrospective Multicentre Study
Source: J Clin Med. 2022 Aug 30;11(17):5101. doi: 10.3390/jcm11175101 (PMC9457026; doi:10.3390/jcm11175101)
Supplement: Supplementary file 1 [file jcm-11-05101-s001.zip › jcm-1845149-supplementary.pdf]

Supplemental Table S1: Time interval between last COVID-19 vaccination and time-point of diagnosis of retinal vascular occlusion (n=410; 321 vaccinated patients and 89 patients with no vaccination; missing data: 11).

Vaccination time point	<2 weeks	2-4 weeks	4-6 weeks	>6 weeks	No vaccination
**All patients**	**70 (21.8%)**	**85 (26.5%)**	**44 (13.7%)**	**122 (38.0%)**	**89**
**CRVO**	22	27	11	38	20
**BRVO**	14	13	10	19	18
**CRAO**	5	9	4	25	13
**BRAO**	15	12	9	17	10
**AION**	14	24	10	23	28

Supplemental Table S2: Characteristics of the study subjects and population-based GHS controls in the case-control study approach.

	**Cases**	**Controls**
**n**	327	327
**Age [years]**	64.8 ± 12.1	64.4±11.6
**Sex [female]**	43.1%	43.1%
**Arterial hypertension [yes]**	63.2%	59.3%
**Diabetes [yes]**	18.3%	11.0%
**Obesity [yes]**	20.2%	21.7%
**COVID-19 vaccination status**		
**Vaccination within the last 4 weeks [yes]**	41.6%	38.2%
**CRVO/BRVO/CRAO/BRAO/AION**	93/63/39/47/85	-/-/-/-/-

CRVO= Central Retinal Vein Occlusion; BRVO= Branch Retinal Vein Occlusion; CRAO= Central Retinal Artery Occlusion; BRAO= Branch Retinal Artery Occlusion; AION= Anterior Ischaemic Optic Neuropathy

Supplemental Table S3: Time-dependent distribution of vaccination stratified for different COVID-19 vaccines prior to onset of symptoms.

	<2 weeks	2-4 weeks	4-6 weeks	>6 weeks
ChadOx1/ *AstraZeneca*	14	21	3	17
BNT162b2/ *BioNTech*	45	54	33	83
mRNA-1273/ *Moderna*	5	5	3	7
Ad26.COV2.S/ *Johnson & Johnson*	6	2	1	2

N=22 missing data on type of vaccination and further n=9 with missing data on date of vaccination

Supplemental Material: Financial Disclosures

- Nicolas Feltgen.: Consultant: Alimera Sciences, Chiesi, Novartis, Roche
  Speaker: Allergan/Abbvie, Alimera, Apellis, Bayer, Heidelberg Engineering, Novartis, Roche
- Thomas Ach: Speaker: Bayer, Novartis, Roche, Apellis; Research grants: Nidek, Novartis.
- Focke Ziemssen: Consultant: Alimera, Allergan/Abbvie, Bayer Healthcare, Boehringer-Ingelheim, Roche/Genentech, NovoNordisk, MSD, Oxurion
  Speaker: Allergan/Abbvie, Bayer Healthcare, CME Health, Novartis, ODOS, Roche
- Carolin Quante: none
- Marc Schargus: Consultant: Allergan/Abbvie, Novartis, iStar Medical
  Speaker: Bayer Healthcare, Allergan Abbvie, Novartis
- Alexander Schuster: holds the professorship for ophthalmic healthcare research endowed by "Stiftung Auge" and financed by "Deutsche Ophthalmologische Gesellschaft" and "Berufsverband der Augenärzte Deutschlands e.V. He receives research support from Allergan/Abbvie, Bayer Healthcare, Heidelberg Engineering, and Novartis.
- Oliver Gross: Consultant for Sanofi US Services Inc./Genzyme/Regulus Therapeutics; Roche; Boehringer Ingelheim; Reata Pharmaceuticals; ONO-Pharmaceutical Co. Ltd.; Codon-X Therapeutics Inc.; Bayer AG; Novartis; Galapagos;
  Advisory committee for Sanofi US Services Inc./Genzyme/Regulus Therapeutics; Roche; Boehringer Ingelheim; Reata Pharmaceuticals
- Alaa Din Abdin: none
- Sabine Aisenbrey: none
- Martin C. Bartram: none
- Marcus Blum: Consultant: Carl Zeiss Meditec, Rheacell Speaker: Allergan/Abbvie, Fielmann
- Claudia Brockmann: Member of Vision Academy, Bayer Healthcare
- Stefan Dithmar: none
- Wilko Friedrichs: none
- Rainer Guthoff: Registration fee reimbursement by Bayer AG and DORC, Speaker Hoffmann La Roche.
- Lars-Olof Hattenbach: Dr. Hattenbach reports grants and personal fees from Novartis Pharma GmbH; grants and personal fees from Bayer AG; personal fees from Pharm Allergan GmbH; grants

and personal fees from Roche; grants from Apellis; and grants from Chengdu Kanghong Biotech Co., Ltd. outside the submitted work.

- Susanne Kaskel-Paul: none
- Ramin Khoramnia: RK reports grants from Chengdu Kanghong; grants, personal fees, and nonfinancial support from Alimera, Bayer, Novartis and Roche, personal fees, and nonfinancial support from Allergan and Heidelberg Engineering outside the submitted work.
- Julian Klaas: receives ongoing speaker honoraria from Novartis GmbH
- Tim U. Krohne, Financial support of research projects: Bayer, Novartis. Recipient of lecture fees: Alimera Sciences, Allergan, Bayer, Heidelberg Engineering, Novartis, Roche. Consultant: Alimera Sciences, Bayer, Novartis, Roche.
- Albrecht Lommatzsch: Consultant: Novartis, Bayer
  Speaker: Bayer vital, Novartis, Roche, Zeiss, Apellis
- Sabine Lueken: none
- Mathias Maier: none
- Lina Nassri: none
- Thien An Nguyen-Dang: none
- Viola Radeck: none
- Saskia Rau: Speaker Allergan/Abbvie, Alimera, Novartis, Bayer
- Johann Roider: Speaker: GlaxoSmithKline, Novartis
- Dirk Sandner: Consultant: Alimera Sciences, Allergan/Abbvie, Bayer Healthcare Novartis, GlaxoSmithKline, Roche
  Speaker: Alimera Sciences, Allergan/Abbvie, Bayer Healthcare, GlaxoSmithKline, Novartis, MSD, Roche, Takeda
- Laura Schmalenberger: none
- Irene Schmidtmann:
- Florian Schubert: none
- Helena Siegel:
- Martin S. Spitzer: Consultant: Allergan/Abbvie, Bayer Healthcare, Neurogene, Novartis,
- Speaker: Allergan/Abbvie, Bayer Healthcare, Boehringer Ingelheim, GSK, Novartis, ODOS, Takeda
- Andreas Stahl: Consultant: Alcon, Apellis, Bayer, Novartis, Roche
  Speaker: Allergan, Bayer, Novartis
- Julia V. Stingl: none
- Felix Treumer: none
- Arne Viestenz: none
- Joachim Wachtlin: Consultant: Novartis, Bayer Speaker: Alcon, Allergan, Bayer, Novartis
- Armin Wolf: Consultant: Alimera, Allergan/Abbvie, Bayer Healthcare, Boehringer-Ingelheim, Roche/Genentech, Santen, Zeiss
  Speaker: Allergan/Abbvie, Bayer Healthcare, Novartis, Optos, Oertli Instruments, Roche, Zeiss
- Julian Zimmermann: none

Supplemental Material: Participating centres

Participating Centres
• Clinic of Ophthalmology, University Medical Centre Goettingen, Goettingen, Germany.<br>• Department of Ophthalmology, University Leipzig, Leipzig, Germany; Centre for Ophthalmology, University Eye Hospital Tübingen, Tübingen, Germany<br>• Clinic of Nephrology and Rheumatology, University Medical Centre Goettingen, Goettingen, Germany.<br>• Department of Ophthalmology, University Hospital Bonn, Bonn, Germany.<br>• Department of Ophthalmology, Vivantes Health Network Ltd, Neukoelln Hospital, Berlin, Germany<br>• Department of Ophthalmology, Hannover Medical School, Hannover, Germany.<br>• Department of Ophthalmology, Helios Hospital Erfurt, Erfurt, Germany.<br>• Department of Ophthalmology, Universitätsmedizin Rostock, Rostock, Germany.<br>• Department of Ophthalmology, Helios HSK Wiesbaden, Wiesbaden, Germany<br>• Charlottenklinik Ophthalmology, Stuttgart, Germany.<br>• Department of Ophthalmology, Faculty of Medicine, University Hospital Duesseldorf, Dusseldorf, Germany.<br>• Department of Ophthalmology, Ludwigshafen Hospital, Ludwigshafen am Rhein, Germany.<br>• Department of Internal Medicine I, Asklepios Klinik Nord, Hamburg, Germany<br>• Department of Ophthalmology, Maerkische Kliniken GmbH, Lüdenscheid, Germany.<br>• The David J Apple Centre for Vision Research, Department of Ophthalmology, University Hospital Heidelberg, Heidelberg, Germany.<br>• Department of Ophthalmology, University Homburg/ Saar, Germany<br>• Department of Ophthalmology, University Hospital, LMU Munich, Germany<br>• Department of Ophthalmology, Faculty of Medicine and University Hospital of Cologne, University of Cologne, Cologne, Germany<br>• Department of Ophthalmology, St. Franziskus-Hospital, Muenster, Germany.<br>• Department of Ophthalmology, University of Luebeck, Luebeck, Germany.<br>• Ophthalmology Department, Hospital rechts der Isar, Technical University of Munich (TUM), Munich, Germany.<br>• Department of Ophthalmology, University Hospital RWTH Aachen, Germany.<br>• Praxis Prof. Laube, Duesseldorf, Germany.<br>• Department of Ophthalmology, University Hospital of Regensburg, Regensburg, Germany.<br>• Department of Ophthalmology, Charité-University Medicine Berlin, Corporate Member of Freie Universität Berlin, Humboldt- Universität zu Berlin and Berlin Institute of Health, Berlin, Germany<br>• Universitätsklinikum Schleswig-Holstein, Klinik für Ophthalmologie, Campus Kiel, Kiel, Germany.

- Department of Ophthalmology, Univ. Hospital Carl Gustav Carus, TU Dresden, Germany.
- Municipal Clinic Braunschweig, Germany.
- Institute of Medical Biostatistics, Epidemiology and Informatics, University Medical Center of the Johannes Gutenberg-University Mainz, Mainz, Germany
- Department of Ophthalmology, Phillips University of Marburg, Marburg, Germany
- Eye Center, Medical Centre-University of Freiburg, Faculty of Medicine, University of Freiburg, Freiburg im Breisgau, Germany.
- Department of Ophthalmology, University Medical Centre Hamburg-Eppendorf, Hamburg, Germany.
- Department of Ophthalmology, University Medical Centre Greifswald, Greifswald, Germany.
- Department of Ophthalmology, University Medical Centre of the Johannes Gutenberg-University Mainz, Mainz, Germany.
- Department of Ophthalmology, Klinikum Kassel, Kassel, Germany
- Department of Ophthalmology, Martin-Luther University Halle-Wittenberg, Halle (Saale) Germany.
- Department of Ophthalmology, Sankt-Gertrauden Krankenhaus, Berlin, Germany and MHB Medizinische Hochschule Brandenburg, Neuruppin, Germany
- Department of Ophthalmology, Ulm University Medical Centre, Ulm, Germany.
- Department of Ophthalmology, University of Muenster Medical Centre, Muenster, Germany.
- Asklepios Augenklinik Nord, Hamburg, Germany
- Department of Ophthalmology, University Hospital Duesseldorf, Heinrich Heine University Duesseldorf, Germany
- Department of Ophthalmology, University Medical Center of the Johannes Gutenberg-University Mainz, Mainz, Germany.
